# Left Atrium Papillary Fibroelastomas: A Cause of Cerebral Emboli

**DOI:** 10.1155/2012/704098

**Published:** 2012-04-29

**Authors:** A. G. Ciss, P. S. Ba, P. A. Dieng, K. Azarnoush, L. Camilleri, E. Geoffroy, A. Innorta, B. Legault, B. Cosserant, C. de Riberolles, M. N'diaye

**Affiliations:** ^1^Service de Chirurgie Thoracique et Cardiovasculaire, Centre Hospitalier National Universitaire de Fann, BP 5035, Dakar, Senegal; ^2^Departement of Cardiovascular Surgery, Hospital University of Clermont-Ferrand, BP 63000, Clermont-Ferrand, France

## Abstract

Papillary fibroelastomas are cardiac benign tumours. Among the benign cardiac tumor, papillary fibroelastomas are reported second after myxomas. Most often diagnosed incidentally, papillary fibroelastomas may embolize to cerebral circulation. Valvular locations are predominant; location in left atrium is rare. In this paper, we present a case of papillary fibroelastoma located in left atrium with symptoms of cerebral embolization. Transoesophageal echocardiography diagnosed a mobile mass. The patient was treated with surgical resection without further embolic complication.

## 1. Introduction

Primary cardiac tumors are rare. In autopsy series their incidence varies between 0.0017 and 0.28%. Papillary fibrolelastoma (PFE) is the third most common benign cardiac tumor after atrial myxomas and lipomas. PFE constitutes 10% of primary cardiac tumors [1]. The majority of PFEs are asymptomatic. Cardiac tumors are a rare cause of cerebral emboli. Most often when diagnosed, PFE may embolize to cerebral circulation. When a patient presents a cerebral embolic event not explained by other cardiovascular or neurological diseases, transthoracic echocardiography (TTE) and transesophageal echocardiography (TEE) are necessary to exclude cardiac tumor.

## 2. Case Report

**Figure 1:**
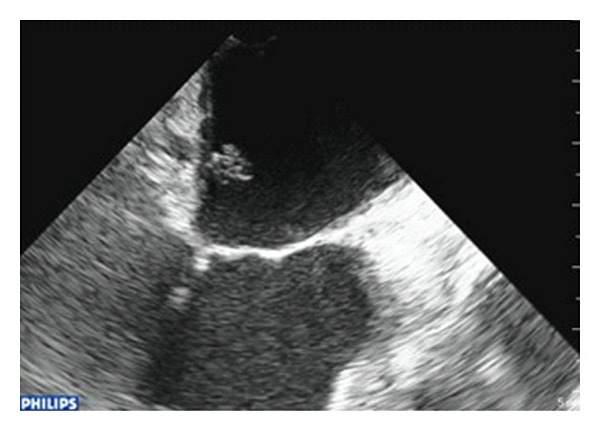
Transesophageal image of the left atrial mass.

**Figure 2:**
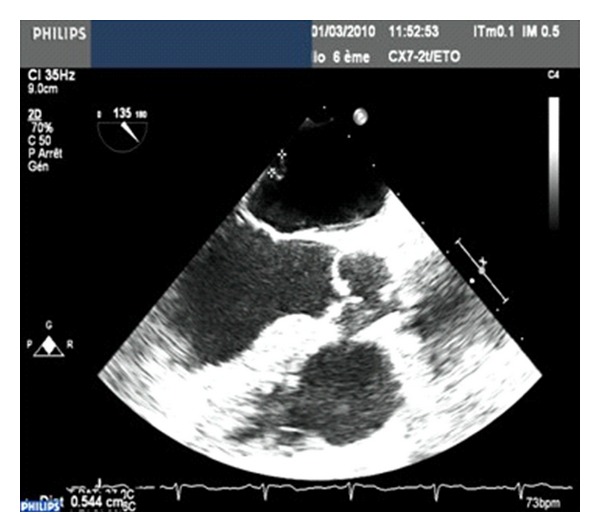
Measurement of two masses in left atrium.

A sixty-four-year-old woman was referred to the department of cardiovascular surgery for the management of ischemic cerebral stroke suspected of cardiac etiology. During the preceding four months, she experienced one episode of transient ischemic cerebral attack. Physical examination was consistent with a monoparesis of left upper extremity and left facial paralysis. No more sign occurred. Cerebral CT scan revealed embolic lesion in the right sylvian artery area. Medical history showed a high blood pressure and hypercholesterolemia. The patient had no prior history of arrhythmia. Electrocardiogram (EKG) and Holter EKG were in sinusal rhythm. Investigations in search of a potential cause of cerebral embolism included a Doppler ultrasound of the carotid arteries which was normal. Transthoracic echocardiography (TTE) (Figure 1) showed an 8 × 8 mm mobile mass attached to the left atrium wall. Transesophageal echocardiography (TEE) (Figure 2) showed a second mass attached to free wall of left atrium (4.3 mm).

The tumors were excised under normothermic cardiopulmonary bypass using ascending aorta and bicaval cannulation. Anterograde blood cardioplegia was used. The left atrium was exposed by extended vertical transatrial septal incision (Guiraudon). Two tumors were found: a 10 mm friable translucide mass was attached between the two pulmonary veins and a 6 mm tumor located in left atrium between right inferior pulmonary vein and the base of anterior mitral leaflet. The left atrial appendage was closed. Mitral leaflet and subvalvular apparatus were inspected and were free of tumors. After bypass arrest a TEE confirmed a left atrium cavity with no tumor. Histological examination showed a papillary fibroelastoma including few fibroblasts, collagenous tissue, and elastic fibers surrounded by mucopolysaccharide acid. The patient had uncomplicated perioperative course and was discharged from hospital on postoperative day 8. At the 6-month followup, the patient was well. She has not experienced any neurological event. Transthoracic and transesophageal echocardiography did not find any recurrent tumour at 6-month followup. 

## 3. Discussion

PFE is a rare primary cardiac tumor. PFEs are predominantly found on the valvular endocardium (80%). Although they may be present anywhere in the heart, but the location in left atrium is rare [2]. Most of PFEs are asymptomatic and are found during autopsy. TTE resolution will not detect tumor as small as 2 mm [3], but is highly precise in larger tumors. Etiology of papillary fibroelastoma is unknown. Multiple theories have been advocated: inflammatory reaction of infection, neoplasms, congenital malformation, or organized thrombi. Two cytogenetic studies [4, 5] showed a structural rearrangement involving chromosomes 5 and 21. A prior study suggested a balanced translocation in both stemline clones in the case, whereas the second case showed unbalanced rearrangement in only the sideline clone [5].

Tumor's location in the heart determines symptoms. Left side location is associated with embolism in systemic circulation which induces cerebral ischemia, myocardial infarction, retinal artery obstruction, renal infarction, and mesenteric or limb ischemia. High potential embolism and high risk of recurrent cerebral ischemia of PFE justify surgical resection [6, 7]. Right-sided friable cardiac tumors can induce recurrent pulmonary embolization and pulmonary hypertension [8]. We believe that all papillary fibroelastoma should be removed surgically, even if the asymptomatic could be exposed to the risk of embolic complications from this benign tumor. There are controversies about the management of PFE; some authors suggest that small asymptomatic tumors should be observed while the larger mobile tumors should be excised [3]. This patient did not have a dilated left atrium. We used the extended vertical transatrial septal incision which resulted in good exposure. After tumor's excision, we closed the left atrial appendage to prevent further thrombus and embolization. The appendage presentation is rare [9].

## 4. Conclusion

 Papillary fibroelastoma is a rare cause of cerebral emboli and should be suspected and ruled out in such clinical presentation.

## References

[1] McAllister HA, Fenoglio JJ (1978). Tumors of the cardiovascular system. *Atlas of Tumor Pathology*.

[2] Gowda RM, Khan IA, Nair CK, Mehta NJ, Vasavada BC, Sacchi TJ (2003). Cardiac papillary fibroelastoma: a comprehensive analysis of 725 cases. *American Heart Journal*.

[3] Sun JP, Asher CR, Yang XS (2001). Clinical and echocardiographic characteristics of papillary fibroelastomas: a retrospective and prospective study in 162 patients. *Circulation*.

[4] Speights VO, Dobin SM, Truss LM (1998). A cytogenetic study of a cardiac papillary fibroelastoma. *Cancer Genetics and Cytogenetics*.

[5] Wachtel M, Heritage DW, Pastore L, Rhee J (2000). Cytogenetic study of a cardiac papillary fibroelastoma. *Cancer Genetics and Cytogenetics*.

[6] McFadden PM, Lacy JR (1987). Intracardiac papillary fibroelastoma: an occult cause of embolic neurologic deficit. *Annals of Thoracic Surgery*.

[7] Topol EJ, Biern RO, Reitz BA (1986). Cardiac papillary fibroelastoma and stroke. Echocardiographic diagnosis and guide to excision. *American Journal of Medicine*.

[8] Gallas MT, Reardon MJ, Reardon PR, DeFelice CA, Raizner AE, Mody DR (1993). Papillary fibroelastoma: a right atrial presentation. *Texas Heart Institute Journal*.

[9] Jablonski-Cohen M, Ohsie L, Bhatti T, Bhatti T, Morris DL (2008). Unusual left atrial appendage mass: atypical presentation of papillary fibroelastoma. *Echocardiography*.

